# Synthesis, crystal structure and Hirshfeld surface analysis of 2-chloro-3-[(*E*)-(2-phenyl­hydrazinyl­idene)meth­yl]quinoline

**DOI:** 10.1107/S2056989019007692

**Published:** 2019-06-07

**Authors:** Soufiane Akhramez, Abderrafia Hafid, Mostafa Khouili, Mohamed Saadi, Lahcen El Ammari, El Mostafa Ketatni

**Affiliations:** a Laboratory of Organic and Analytical Chemistry, University Sultan Moulay Slimane, Faculty of Science and Technology, PO Box 523, Beni-Mellal, Morocco; b Laboratoire de Chimie Appliquée des Matériaux, Centre Sciences des Matériaux, Faculty of Sciences, Mohammed V University in Rabat, Avenue Ibn Batouta, BP 1014, Rabat, Morocco

**Keywords:** crystal structure, quinoline hydrazine, phenyl hydrazine, C—H⋯π inter­action, weak N—H⋯π inter­action, Hirshfeld surface analysis

## Abstract

A new quinoline-based hydrazone, C_16_H_12_ClN_3_, was synthesized by a condensation reaction of 2-chloro-3-formyl­quinoline with phenyl­hydrazine. The quinoline ring system is essentially planar (r.m.s. deviation = 0.012 Å), and forms a dihedral angle of 8.46 (10)° with the phenyl ring. The mol­ecule adopts an *E* configuration with respect to the central C=N bond. In the crystal, mol­ecules are linked by a C—H⋯π-phenyl inter­action, forming zigzag chains propagating along the [10] direction. The N—H hydrogen atom does not participate in hydrogen bonding but is directed towards the phenyl ring of an adjacent mol­ecule, so linking the chains *via* weak N—H⋯π inter­actions to form of a three-dimensional structure. The Hirshfeld surface analysis of the crystal structure indicates that the most important contributions to the crystal packing are from H⋯H (35.5%), C⋯H/H⋯C (33.7%), Cl⋯H/H⋯Cl (12.3%), N⋯H/H⋯N (9.5%) contacts.

## Abstract 2

A new quinoline-based hydrazone compound, has been synthesized by a condensation reaction of 2-chloro-3-formyl­quinoline with phenyl­hydrazine. The quinoline ring system is essentially planar (r.m.s. deviation = 0.012 Å), and forms a dihedral angle of 8.46 (10)° with the phenyl ring substituent.

## Chemical context   

Quinoline hydrazones are important classes of organic compounds that have long attracted attention because of their potential biological and pharmacological properties. They were conventionally prepared by a condensation reaction of the carbonyl compounds with hydrazines. A number of compounds incorporating the quinolinic heterocycle and a hydrazone have been synthesized and tested for their potential as anti­tumor agents (Erguc *et al.*, 2018 ▸; Mandewale *et al.*, 2017 ▸). Hydrazono-quinoline derivatives have been incorp­orated in many synthetic heterocyclic compounds in order to enhance the cytotoxic activity (Bingul *et al.*, 2016 ▸). Some of these derivatives may have anti-tuberculosis activity *in vitro* against various strains of *Mycobacterium* (Eswaran *et al.*, 2010*a* ▸,*b* ▸, 2009 ▸). Others have been studied as anti­bacterial agents (Desai *et al.*, 2014 ▸; Vlahov *et al.*, 1990 ▸) and anti­malarials (Vandekerckhove & D’hooghe, 2015 ▸; Lyon *et al.*, 1999 ▸; Nayak *et al.*, 2016 ▸; Hamama *et al.*, 2018 ▸; Chavan *et al.*, 2016 ▸).
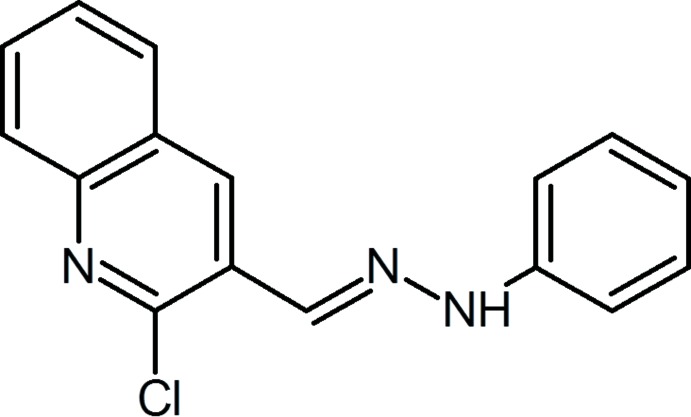


**Figure 1:**
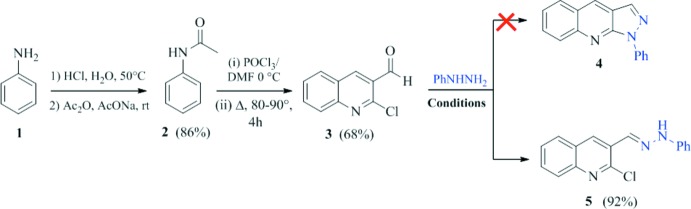
Reaction scheme: condensation of 2-chloro-3-formyl­quinoline with phenyl­hydrazine.

In an attempt to find novel bioactive cytotoxic mol­ecules, we have synthesized a series of quinoline-3-carbo­nitrile and 2-chloro­quinoline derivatives by the reaction mechanism illus­trated in Fig. 1 ▸. A similar synthesis has been reported in the literature (Korcz *et al.*, 2018 ▸).

The structure of the title compound **5**, has been elucidated using ^1^H and ^13^C NMR spectroscopy and X-ray diffraction analysis.

## Structural commentary   

**Figure 2:**
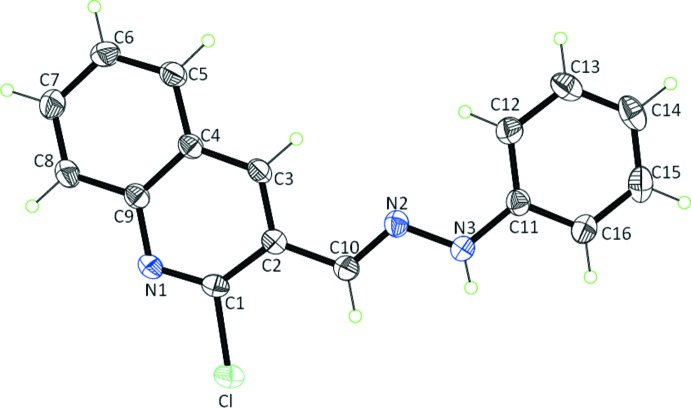
The mol­ecular structure of compound **5** with the atom labelling. Displacement ellipsoids are drawn at the 30% probability level.

Compound **5** was prepared by a condensation reaction of 2-chloro-3-formyl­quinoline with phenyl­hydrazine. It crystallizes in the monoclinic space group *Cc*. It is composed of a phenyl ring and a quinoline ring system linked by a –CH=N—NH– spacer (Fig. 2 ▸), and adopts an *E* configuration relative to the hydrazonic N2=C10 bond [1.277 (3) Å].

The quinoline moiety is very slightly twisted, as indicated by the dihedral angle of 0.99 (10)° between the C1–C6 and N1/C1–C4/C9 rings. The phenyl ring (C11–C16) makes a dihedral angle of 8.49 (9)° with the mean plane of the quinoline ring system. The C1—Cl1 bond length of 1.750 (2) Å is in good agreement with the value of 1.756 (2) Å reported for a related structure, *viz*. (*E*)-1-[(2-chloro­quinolin-3-yl)methyl­ene]-2-(4-methyl­phen­yl)hydrazine, also known as 2-chloro-3-{[(4-methyl­phen­yl)hydrazono]meth­yl)quinoline} (Kumara *et al.*, 2016 ▸).

## Supra­molecular features   

**Figure 3:**
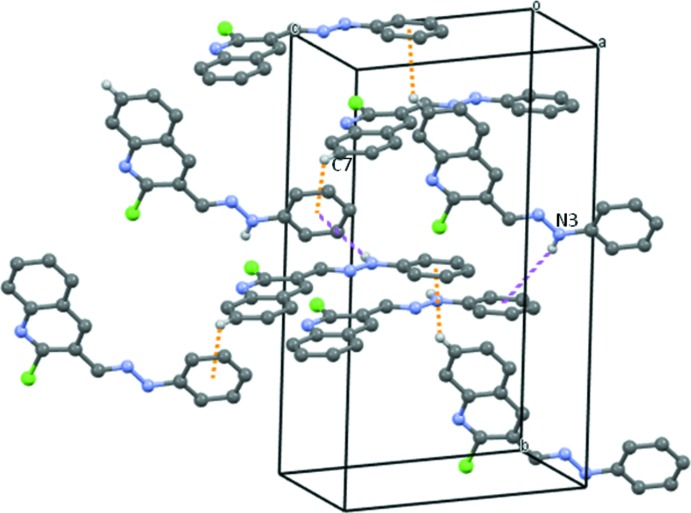
Chain of mol­ecules of compound **5** linked by C—H⋯π and N—H⋯π inter­actions (Table 1 ▸), shown respectively, as dotted orange and purple dashed lines. For clarity, H atoms not involved in these inter­actions have been omitted.

**Figure 4:**
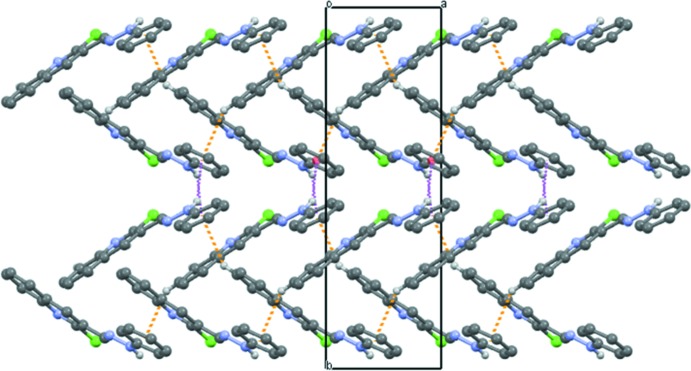
A view along the *c* axis of the crystal packing of compound **5**. H atoms not involved in the C—H⋯π and N—H⋯π inter­actions (dotted orange and purple dashed lines, respectively) have been omitted for clarity.

In the crystal of compound **5**, mol­ecules are linked by a C—H⋯π-phenyl inter­action (Table 1 ▸), with an H⋯centroid distance of 2.97 Å, forming zigzag chains propagating along the [10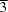] direction, as shown in Fig. 3 ▸. The NH group of the hydrazone moiety does not form a hydrogen bond, but is directed towards the phenyl ring of an adjacent mol­ecule, so linking the chains *via* a weak N—H⋯π inter­action (Table 1 ▸), to form of a supra­molecular three-dimensional structure (Fig. 4 ▸). There are no other significant inter­molecular contacts shorter than those of the sum of the van der Waals radii of the individual atoms (*PLATON*; Spek, 2009 ▸).

## Hirshfeld surface analysis and two-dimensional fingerprint plots   

**Figure 5:**
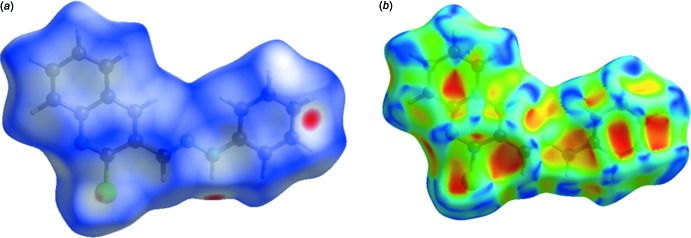
A view of the Hirshfeld surface of compound **5** mapped over (*a*) *d*_norm_ and (*b*) shape index.

In order to visualize the role of weak inter­molecular contacts in the crystal of compound **5**, a Hirshfeld surface (HS) analysis (Spackman & Jayatilaka, 2009 ▸) was carried out and the associated two-dimensional fingerprint plots (McKinnon *et al.*, 2007 ▸) generated using *CrystalExplorer17.5* (Turner *et al.*, 2017 ▸). The three-dimensional *d*_norm_ surface of **5** is shown in Fig. 5 ▸ with a standard surface resolution and a fixed colour scale of −0.1805 to 1.0413 a.u. The darkest red spots on the Hirshfeld surface indicate contact points with atoms participating in inter­molecular C—H⋯π and N—H⋯π inter­actions that involve C7—H7 and N3—H3*N* and the phenyl substituent (Table 1 ▸).

**Figure 6:**
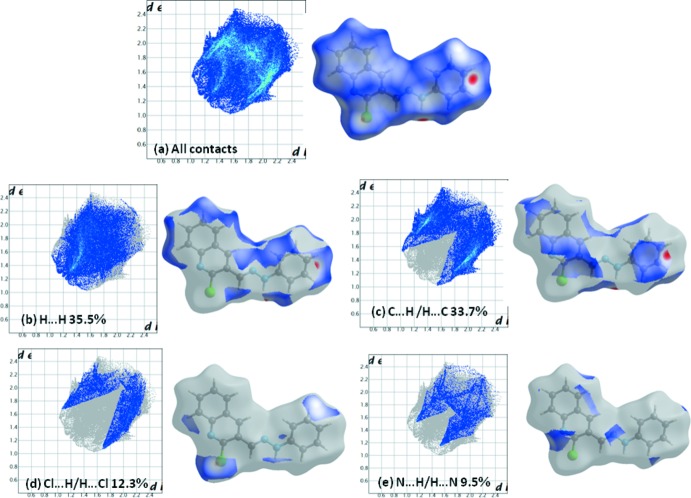
The overall two-dimensional fingerprint plot for compound **5**, and those delineated into: (*b*) H⋯H (35.5%), (*c*) C⋯H/H⋯C (33.7%), (*d*) Cl⋯H/H.·Cl (12.3%) and (*e*) N⋯H/H⋯N (9.5%) contacts.

As illustrated in Fig. 6 ▸, the corresponding fingerprint plots for compound **5** have characteristic pseudo-symmetric wings along the *d*_e_ and *d*_i_ diagonal axes. The presence of C—H⋯π and N—H⋯π inter­actions in the crystal are indicated by the pair of characteristic wings in the fingerprint plot delineated into C⋯H/H⋯C (Fig. 6 ▸ *c*) and N⋯H/H⋯N (Fig. 6 ▸ *e*) contacts (33.7 and 9.5% contributions, respectively, to the Hirshfeld surface). As shown in Fig. 6 ▸ *b*, the most widely scattered points in the fingerprint plot are related to H⋯H contacts, which make a contribution of 35.5% to the Hirshfeld surface. There are also Cl⋯H/H⋯Cl (12.3%; Fig. 6 ▸ *d*) and N⋯H/H⋯N (9.5%; Fig. 6 ▸ *e*) contacts, with smaller contributions from C⋯C (3.5%), Cl⋯N (2.3%), C⋯N (2.2%) and C⋯Cl (1.1%) contacts.

## Database survey   

**Figure 7:**
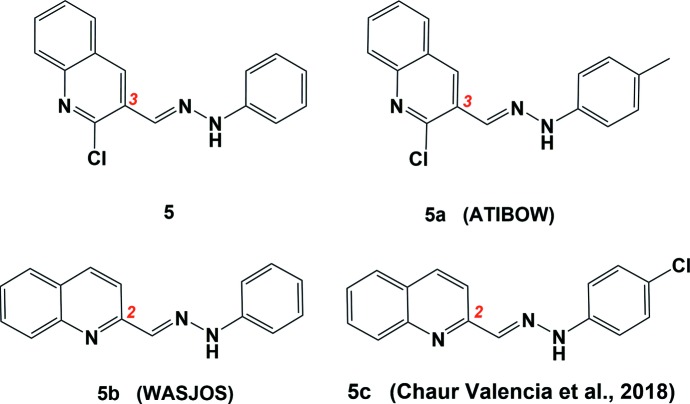
Structures of some related quinoline-hydrazine compounds.

A search of the Cambridge Structural Database (CSD, version 5.40, update February 2019; Groom *et al.*, 2016 ▸) using the hydrazinylidenemethyl quinoline system (Fig. 7 ▸) as the main skeleton revealed the presence of three similar structures to the title compound. One compound, (*E*)-2-chloro-3-{[2-(*p*-tol­yl)hydrazineyl­idene]meth­yl}quinoline (**5a**) (CSD refcode ATIBOW; Kumara *et al.*, 2016 ▸) has the C=N—N linkage with quinoline at position-3, as in the title compound **5**. Two compounds have the C=N—N linkage with quinoline at position-2, *viz*. (*E*)-2-[(2-phenyl­hydrazineyl­idene)meth­yl]quinoline (**5b**) (WASJOS; Mukherjee *et al.*, 2014 ▸) and (*E*)-2-{[2-(4-chloro­phen­yl)hydrazineyl­idene]meth­yl}quinoline (**5c**) (Chaur Valencia *et al.*, 2018 ▸), as shown in Fig. 7 ▸. Table 2 ▸ presents a comparison between the principal bond lengths and angles of compound **5** and the related structures. The bond lengths in the hydrazonic linkage –C=N—N– remain almost unaltered in all four compounds, as do the C—C=N—N torsion angles.

## Synthesis and crystallization   

The multi-step reactions leading to the synthesis of the title compound **5** are illustrated in Fig. 1 ▸. Details of the syntheses of compounds **2**, **3** and **5** are given below.

2-Chloro­quinoline-3-carbaldehyde (**3**) was synthesized from acetyl­ated aniline (**2**), according to a Vilsmeier–Haack reaction, either by conventional methods (Ramesh *et al.*, 2008 ▸; Rajakumar & Raja, 2010 ▸), using microwaves (Mogilaiah *et al.*, 2002 ▸) or ultrasonic irradiation (Ali *et al.*, 2002 ▸).

In a first step, we tried a simple reaction of 2-chloro­quinoline-3-carbaldehyde (**3**) and phenyl hydrazine in ethanol at room temperature or with heating to synthesize a new pyrazolo-quinoline derivative, 1-phenyl-1*H*-pyrazolo[3,4-*b*]quinoline (**4**). This was by a simple and different method from that described in the literature (Hamama *et al.*, 2018 ▸). Unfortunately, the reaction did not take the desired route and led to the formation of the title compound **5**, 2-chloro-3-[(*E*)-(2-phenyl­hydrazinyl­idene)meth­yl]quinoline, resulting from the attack of the nitro­gen of hydrazine on the aldehyde at position 3 of quinoline.

The reaction conditions for the synthesis of compound **5** were optimized by changing the solvent, the catalyst and the temperature. The best yield of 92% was obtained by the conventional method, *viz*. refluxing in ethanol for 10 min and without a catalyst. In the ^1^H NMR spectra of this hydrazone quinoline, the single resonance for the proton of the –N(H)N=group is observed at δ = 12.01 ppm, whereas the corresponding amide N=CH proton appears as a broad singlet at 8.45 ppm. The spectra show that the chemical shifts of the protons on the aryl group have been assigned correctly. The structure of this hydrazono-quinoline, **5**, was confirmed by the single-crystal X-ray diffraction study.

**Synthesis of** ***N*** **-phenyl­acetamide (2)**:

To a 500 ml flask containing 250 ml of water and 25% hydro­chloric acid (15 ml, 0.108 mol), aniline (9.75 ml, 0.108 mol) was added. The reaction mixture was heated at 323 K for 10 min. Then, and at room temperature, acetic anhydride (10.3 ml, 0.108 mol) and sodium acetate (16.4 g, 0.2 mol) were added. The mixture was stirred for 20 min. The product obtained was filtered off and then dried, giving a white solid (yield 86%, m.p. 384–386 K).

^1^H NMR (300 Hz, CDCl_3_): δ (ppm) 7.42 (1H, *s*, NH), 7.77–7.22 (5H, *m*, HAr), 2,21 (3H, *s*, CH_3_); ^13^C NMR (75 Hz, CDCl_3_): δ (ppm) 169.9 (CO), 140.0 (C), 129.6 (2C), 128.7 (C), 122.3 (2CH), 20.9 (CH_3_).

**Synthesis of 2-chloro­quinoline-3-carbaldehyde (3)**:

Phospho­rus oxychloride (POCl_3_) (35 ml, 374 mmol) was added dropwise with magnetic stirring at 273 K, to anhydrous *N*,*N*-di­methyl­formamide (DMF) (10 ml, 135 mmol) in a double-necked flask. Once the addition was complete, the temperature was allowed to rise and the reaction mixture was left stirring for 30 min. Acetanilide **2** (7.29 g, 54 mmol) was then added and the reaction mixture was heated at 348 K for 4 h. Subsequently and at room temperature, the reaction mixture was poured in small portions into an Erlenmeyer flask containing a mixture of ice/water (200 ml) maintained with magnetic stirring. The precipitate formed was filtered and then washed with water (100 ml). Compound **3** was obtained as a yellow solid (yield 68%, m.p. 418–420 K).

^1^HNMR (300 Hz, CDCl_3_): δ (ppm) 10.59 (1H, *s*, CHO), 8.80 (1H, *s*), 8.07–7.66 (4H, *m*, HAr); ^13^CNMR (75 Hz, CDCl_3_): δ (ppm) 189.2 (CHO), 150.1 (C), 149.5 (C), 140.2 (CH), 133.6 (CH), 129.7 (C), 128.5 (CH), 128.1 (CH), 126.4 (CH), 126.3 (C).

**Synthesis of 2-chloro-3-[(** ***E*** **)-(2-phenyl­hydrazinyl­idene)meth­yl]quinoline (5)**:

To a solution of 2-chloro­quinoline-3-carbaldehyde (**3**) (191.0 mg, 1 mmol) in ethanol was added phenyl­hydrazine (0.99 ml, 1 mmol). The mixture was stirred and refluxed for 10 min. The precipitate that formed was filtered, then washed repeatedly with diethyl ether. Subsequently, the precipitate was dissolved in pure ethanol. Pale-brown block-like crystals were obtained by slow evaporation of this ethano­lic solution at room temperature. The crystals were then dried under vacuum (yield 92%, m.p. 429–429 K).

^1^HNMR (300 Hz, CDCl_3_): 12.01 (*s*, 1H, NH), 8.97 (*s*, 1H, H_quinoline_), 8.45 (*s*, 1H, N=CH), 7.90–8.00 (*m*, 4H, Ar-H), 7.62 (*d*, *J* = 8.3 Hz, 2H, Ar-H), 7.33 (*d*, *J* = 7.9 Hz, 2H, Ar-H), 7.06 (*t*, *J* = 7.9 Hz, 1H, Ar-H); ^13^CNMR (75 Hz, CDCl_3_): 121.8, 127.8 (three overlapping signals), 154.1 (CCl), 147.4 (C), 146.3 (C), 135.8 (CN), 133.4 (C), 131.8 (C), 129.9 (2C), 129.5(C), 128.8 (C), 128.0 (C), 127.7 (C), 126.6 (C), 124.1 (C), 122.7 (C), 116.6 (2C).

## Refinement   

Crystal data, data collection and structure refinement details are summarized in Table 3 ▸. All H atoms could be located in a difference-Fourier map. During refinement they were placed in calculated positions and treated as riding: N—H = 0.86 Å, C—H = 0.93 Å with *U*_iso_(H) = 1.2*U*_eq_(N,C).

## Supplementary Material

Crystal structure: contains datablock(s) I, Gobal. DOI: 10.1107/S2056989019007692/su5497sup1.cif

Structure factors: contains datablock(s) I. DOI: 10.1107/S2056989019007692/su5497Isup2.hkl

CCDC reference: 1918954

Additional supporting information:  crystallographic information; 3D view; checkCIF report

## Figures and Tables

**Table 1:** Hydrogen-bond geometry (Å, °) *Cg* is the centroid of the C11–C16 ring.

*D*—H⋯*A*	*D*—H	H⋯*A*	*D*⋯*A*	*D*—H⋯*A*
C7—H7⋯*Cg* ^i^	0.93	2.97	3.700 (3)	136
N3—H3*N*⋯*Cg* ^ii^	0.86	3.30	4.060	149

Symmetry codes: (i) 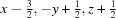; (ii) 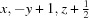.

**Table 2:** Comparison of main bond lengths (Å) and C—C=N—N torsion angles (°) in compound **5** and the related structures **5a**, **5b** and **5c**

Compound	C2—C10	C10=N2	N2—N3	N3—C11	C—C=N—N
**5**	1.461 (3)	1.277 (3)	1.349 (3)	1.391 (3)	−177.79 (19)
**5a**	1.468	1.282	1.354	1.400	−178.40
**5b**	1.452	1.289	1.350	1.393	−179.10
**5c**	1.456	1.279	1.348	1.389	179.10

Notes: **5** this study; **5a** Kumara *et al.* (2016 ▸); **5b** Mukherjee *et al.* (2014 ▸); **5c** Chaur Valencia *et al.* (2018 ▸).

**Table 3:** Experimental details

Crystal data	
Chemical formula	C_16_H_12_ClN_3_
*M*_r_	281.74
Crystal system, space group	Monoclinic, *C* *c*
Temperature (K)	296
*a*, *b*, *c* (Å)	6.2114 (4), 19.4553 (11), 11.2520 (7)
β (°)	91.883 (2)
*V* (Å^3^)	1359.01 (14)
*Z*	4
Radiation type	Mo *K*α
μ (mm^−1^)	0.27
Crystal size (mm)	0.35 × 0.26 × 0.20

Data collection	
Diffractometer	Bruker D8 VENTURE Super DUO
Absorption correction	Multi-scan (*SADABS*; Bruker, 2016 ▸)
*T*_min_, *T*_max_	0.678, 0.746
No. of measured, independent and observed [*I* > 2σ(*I*)] reflections	21613, 2981, 2712
*R*_int_	0.029
(sin θ/λ)_max_ (Å^−1^)	0.641

Refinement	
*R*[*F* ^2^ > 2σ(*F* ^2^)], *wR*(*F* ^2^), *S*	0.029, 0.072, 1.04
No. of reflections	2981
No. of parameters	182
No. of restraints	2
H-atom treatment	H-atom parameters constrained
Δρ_max_, Δρ_min_ (e Å^−3^)	0.15, −0.14
Absolute structure	Flack *x* determined using 1200 quotients [(*I* ^+^)−(*I* ^−^)]/[(*I* ^+^)+(*I* ^−^)] (Parsons *et al.*, 2013 ▸)
Absolute structure parameter	0.023 (15)

Computer programs: *APEX3* and *SAINT-Plus* (Bruker, 2016 ▸), *SHELXS2014* (Sheldrick, 2008 ▸), *SHELXL2014* (Sheldrick, 2015 ▸), *ORTEP-3 for Windows* (Farrugia, 2012 ▸), *Mercury* (Macrae *et al.*, 2008 ▸), *PLATON* (Spek, 2009 ▸) and *publCIF* (Westrip, 2010 ▸).

## supplementary crystallographic information

### Crystal data

**Table:**

C_16_H_12_ClN_3_	*F*(000) = 584
*M**_r_* = 281.74	*D*_x_ = 1.377 Mg m^−^^3^
Monoclinic, *C**c*	Mo *K*α radiation, λ = 0.71073 Å
*a* = 6.2114 (4) Å	Cell parameters from 2981 reflections
*b* = 19.4553 (11) Å	θ = 2.8–27.1°
*c* = 11.2520 (7) Å	µ = 0.27 mm^−^^1^
β = 91.883 (2)°	*T* = 296 K
*V* = 1359.01 (14) Å^3^	Block, brown
*Z* = 4	0.35 × 0.26 × 0.20 mm

### Data collection

**Table:**

Bruker D8 VENTURE Super DUO diffractometer	2981 independent reflections
Radiation source: INCOATEC IµS micro-focus source	2712 reflections with *I* > 2σ(*I*)
HELIOS mirror optics monochromator	*R*_int_ = 0.029
Detector resolution: 10.4167 pixels mm^-1^	θ_max_ = 27.1°, θ_min_ = 2.8°
φ and ω scans	*h* = −7→7
Absorption correction: multi-scan (SADABS; Bruker, 2016)	*k* = −24→24
*T*_min_ = 0.678, *T*_max_ = 0.746	*l* = −14→14
21613 measured reflections	

### Refinement

**Table:**

Refinement on *F*^2^	Hydrogen site location: inferred from neighbouring sites
Least-squares matrix: full	H-atom parameters constrained
*R*[*F*^2^ > 2σ(*F*^2^)] = 0.029	*w* = 1/[σ^2^(*F*_o_^2^) + (0.0402*P*)^2^ + 0.2303*P*] where *P* = (*F*_o_^2^ + 2*F*_c_^2^)/3
*wR*(*F*^2^) = 0.072	(Δ/σ)_max_ < 0.001
*S* = 1.03	Δρ_max_ = 0.15 e Å^−^^3^
2981 reflections	Δρ_min_ = −0.14 e Å^−^^3^
182 parameters	Extinction correction: (SHELXL2014; Sheldrick, 2015), Fc^*^=kFc[1+0.001xFc^2^λ^3^/sin(2θ)]^-1/4^
2 restraints	Extinction coefficient: 0.0046 (13)
Primary atom site location: structure-invariant direct methods	Absolute structure: Flack *x* determined using 1200 quotients [(*I*^+^)-(*I*^-^)]/[(*I*^+^)+(*I*^-^)] (Parsons *et al.*, 2013)
Secondary atom site location: difference Fourier map	Absolute structure parameter: 0.023 (15)

### Special details

**Table:**

Geometry. All esds (except the esd in the dihedral angle between two l.s. planes) are estimated using the full covariance matrix. The cell esds are taken into account individually in the estimation of esds in distances, angles and torsion angles; correlations between esds in cell parameters are only used when they are defined by crystal symmetry. An approximate (isotropic) treatment of cell esds is used for estimating esds involving l.s. planes.

### Fractional atomic coordinates and isotropic or equivalent isotropic displacement parameters (Å^2^)

**Table:**

	*x*	*y*	*z*	*U*_iso_*/*U*_eq_	
Cl1	0.50087 (11)	0.42496 (3)	0.50091 (7)	0.0554 (2)	
N1	0.1727 (3)	0.34866 (11)	0.44617 (16)	0.0421 (4)	
N2	0.6213 (3)	0.40817 (9)	0.12487 (17)	0.0419 (4)	
N3	0.8016 (3)	0.43842 (10)	0.08642 (18)	0.0463 (5)	
H3N	0.8814	0.4624	0.1345	0.056*	
C1	0.3350 (3)	0.37935 (11)	0.39999 (19)	0.0393 (5)	
C2	0.3882 (3)	0.37981 (11)	0.27845 (18)	0.0371 (5)	
C3	0.2499 (4)	0.34346 (12)	0.20400 (18)	0.0395 (5)	
H3	0.2744	0.3424	0.1229	0.047*	
C4	0.0728 (4)	0.30794 (11)	0.24785 (18)	0.0379 (5)	
C5	−0.0709 (4)	0.26905 (12)	0.1754 (2)	0.0470 (5)	
H5	−0.0510	0.2665	0.0940	0.056*	
C6	−0.2389 (4)	0.23516 (13)	0.2241 (2)	0.0511 (6)	
H6	−0.3328	0.2096	0.1756	0.061*	
C7	−0.2712 (5)	0.23870 (13)	0.3473 (3)	0.0544 (6)	
H7	−0.3850	0.2149	0.3798	0.065*	
C8	−0.1374 (4)	0.27659 (13)	0.4190 (2)	0.0498 (6)	
H8	−0.1619	0.2794	0.4999	0.060*	
C9	0.0384 (4)	0.31169 (11)	0.37146 (19)	0.0394 (5)	
C10	0.5788 (4)	0.41442 (11)	0.2346 (2)	0.0421 (5)	
H10	0.6671	0.4404	0.2855	0.051*	
C11	0.8577 (4)	0.43052 (11)	−0.0315 (2)	0.0405 (5)	
C12	0.7277 (4)	0.39628 (13)	−0.1134 (2)	0.0487 (5)	
H12	0.5980	0.3773	−0.0905	0.058*	
C13	0.7903 (5)	0.39014 (15)	−0.2298 (2)	0.0574 (7)	
H13	0.7017	0.3670	−0.2847	0.069*	
C14	0.9814 (5)	0.41769 (13)	−0.2655 (2)	0.0582 (7)	
H14	1.0218	0.4137	−0.3441	0.070*	
C15	1.1114 (5)	0.45108 (15)	−0.1835 (3)	0.0620 (7)	
H15	1.2414	0.4697	−0.2067	0.074*	
C16	1.0518 (5)	0.45757 (15)	−0.0663 (2)	0.0563 (7)	
H16	1.1420	0.4800	−0.0113	0.068*	

### Atomic displacement parameters (Å^2^)

**Table:**

	*U*^11^	*U*^22^	*U*^33^	*U*^12^	*U*^13^	*U*^23^
Cl1	0.0558 (3)	0.0700 (4)	0.0398 (3)	−0.0078 (3)	−0.0075 (2)	−0.0099 (3)
N1	0.0487 (10)	0.0489 (10)	0.0288 (8)	−0.0018 (8)	0.0014 (8)	−0.0004 (7)
N2	0.0435 (11)	0.0417 (10)	0.0407 (10)	−0.0026 (8)	0.0042 (8)	0.0034 (8)
N3	0.0471 (11)	0.0510 (11)	0.0411 (10)	−0.0138 (9)	0.0049 (8)	−0.0024 (8)
C1	0.0438 (12)	0.0401 (11)	0.0336 (10)	0.0036 (9)	−0.0052 (9)	−0.0029 (9)
C2	0.0416 (12)	0.0352 (11)	0.0345 (11)	0.0044 (9)	0.0015 (9)	0.0009 (8)
C3	0.0474 (12)	0.0425 (12)	0.0290 (10)	0.0001 (9)	0.0052 (9)	−0.0021 (8)
C4	0.0453 (12)	0.0349 (10)	0.0335 (10)	0.0029 (9)	0.0025 (9)	0.0002 (8)
C5	0.0574 (14)	0.0469 (12)	0.0368 (12)	−0.0040 (11)	0.0024 (10)	−0.0047 (10)
C6	0.0566 (14)	0.0465 (14)	0.0499 (15)	−0.0112 (11)	−0.0021 (12)	−0.0065 (10)
C7	0.0541 (15)	0.0536 (14)	0.0561 (15)	−0.0123 (12)	0.0110 (12)	0.0013 (12)
C8	0.0575 (15)	0.0561 (15)	0.0361 (12)	−0.0054 (12)	0.0092 (10)	0.0025 (11)
C9	0.0459 (12)	0.0379 (11)	0.0343 (10)	0.0020 (9)	0.0016 (9)	0.0016 (8)
C10	0.0452 (12)	0.0426 (11)	0.0385 (11)	−0.0024 (10)	0.0011 (9)	−0.0009 (9)
C11	0.0454 (12)	0.0364 (12)	0.0401 (12)	−0.0003 (9)	0.0050 (9)	0.0038 (8)
C12	0.0464 (13)	0.0539 (13)	0.0457 (13)	−0.0040 (11)	−0.0004 (10)	0.0031 (10)
C13	0.0755 (19)	0.0575 (16)	0.0388 (13)	0.0056 (14)	−0.0042 (12)	0.0008 (11)
C14	0.084 (2)	0.0474 (14)	0.0441 (13)	0.0173 (14)	0.0182 (13)	0.0101 (11)
C15	0.0650 (18)	0.0549 (15)	0.0677 (18)	−0.0024 (13)	0.0277 (15)	0.0071 (13)
C16	0.0563 (15)	0.0560 (15)	0.0572 (16)	−0.0156 (12)	0.0113 (12)	−0.0022 (12)

### Geometric parameters (Å, º)

**Table:**

Cl1—C1	1.750 (2)	C6—H6	0.9300
N1—C1	1.295 (3)	C7—C8	1.356 (4)
N1—C9	1.369 (3)	C7—H7	0.9300
N2—C10	1.277 (3)	C8—C9	1.408 (3)
N2—N3	1.349 (3)	C8—H8	0.9300
N3—C11	1.391 (3)	C10—H10	0.9300
N3—H3N	0.8600	C11—C12	1.377 (3)
C1—C2	1.417 (3)	C11—C16	1.384 (3)
C2—C3	1.376 (3)	C12—C13	1.384 (3)
C2—C10	1.461 (3)	C12—H12	0.9300
C3—C4	1.402 (3)	C13—C14	1.374 (4)
C3—H3	0.9300	C13—H13	0.9300
C4—C5	1.409 (3)	C14—C15	1.370 (5)
C4—C9	1.416 (3)	C14—H14	0.9300
C5—C6	1.365 (4)	C15—C16	1.387 (4)
C5—H5	0.9300	C15—H15	0.9300
C6—C7	1.409 (4)	C16—H16	0.9300
			
C1—N1—C9	117.55 (18)	C7—C8—H8	119.8
C10—N2—N3	117.97 (19)	C9—C8—H8	119.8
N2—N3—C11	119.62 (19)	N1—C9—C8	119.1 (2)
N2—N3—H3N	120.2	N1—C9—C4	121.43 (19)
C11—N3—H3N	120.2	C8—C9—C4	119.5 (2)
N1—C1—C2	126.8 (2)	N2—C10—C2	118.6 (2)
N1—C1—Cl1	115.01 (16)	N2—C10—H10	120.7
C2—C1—Cl1	118.15 (17)	C2—C10—H10	120.7
C3—C2—C1	115.04 (19)	C12—C11—C16	119.4 (2)
C3—C2—C10	121.86 (19)	C12—C11—N3	122.1 (2)
C1—C2—C10	123.08 (19)	C16—C11—N3	118.4 (2)
C2—C3—C4	121.36 (19)	C11—C12—C13	119.9 (2)
C2—C3—H3	119.3	C11—C12—H12	120.0
C4—C3—H3	119.3	C13—C12—H12	120.0
C3—C4—C5	123.4 (2)	C14—C13—C12	121.0 (3)
C3—C4—C9	117.76 (19)	C14—C13—H13	119.5
C5—C4—C9	118.9 (2)	C12—C13—H13	119.5
C6—C5—C4	120.4 (2)	C15—C14—C13	118.9 (2)
C6—C5—H5	119.8	C15—C14—H14	120.5
C4—C5—H5	119.8	C13—C14—H14	120.5
C5—C6—C7	120.4 (2)	C14—C15—C16	120.9 (3)
C5—C6—H6	119.8	C14—C15—H15	119.5
C7—C6—H6	119.8	C16—C15—H15	119.5
C8—C7—C6	120.5 (2)	C11—C16—C15	119.8 (3)
C8—C7—H7	119.8	C11—C16—H16	120.1
C6—C7—H7	119.8	C15—C16—H16	120.1
C7—C8—C9	120.4 (2)		

### Hydrogen-bond geometry (Å, º)

*Cg* is the centroid of the C11–C16 ring.

**Table:**

*D*—H···*A*	*D*—H	H···*A*	*D*···*A*	*D*—H···*A*
C7—H7···*Cg*^i^	0.93	2.97	3.700 (3)	136
N3—H3*N*···*Cg*^ii^	0.86	3.30	4.060	149

Symmetry codes: (i) *x*−3/2, −*y*+1/2, *z*+1/2; (ii) *x*, −*y*+1, *z*+1/2.
